# The impact of positive airway pressure on midface growth: a literature review^☆^

**DOI:** 10.1016/j.bjorl.2020.05.010

**Published:** 2020-06-15

**Authors:** Rita Catia Brás Bariani, Thais Moura Guimarães, Mario Cappellette, Gustavo Moreira, Reginaldo Raimundo Fujita

**Affiliations:** a Universidade Federal de São Paulo (Unifesp), Departamento de Otorrinolaringologia e Cirurgia de Cabeça e Pescoço, São Paulo, SP, Brazil; b Universidade Federal de São Paulo (Unifesp), Departamento de Psicobiologia, São Paulo, SP, Brazil

**Keywords:** Sleep apnea syndromes, Sleep apnea, obstructive, Continuous positive airway pressure, Ventilation, Síndromes da apneia do sono, Apneia do sono, obstrutiva, Pressão positiva contínua nas vias aéreas, Ventilação

## Abstract

**Introduction:**

The treatment of obstructive sleep apnea with positive airway pressure in children is restricted due to concerns that it could affect maxilla growth over time.

**Objective:**

To undertake a systematic review of the literature about the long-term impact of using a positive airway pressure mask on the midface in growing individuals.

**Methods:**

The literature search was conducted in September 2019 using the keywords (“long-term” OR “long term” OR “side effects” OR longitudinal) AND (children OR child OR preschool OR adolescents OR adolescent OR infant OR infants) AND (craniofacial OR “mid-face” OR midface OR midfacial OR facial OR maxillary) AND (“airway pressure” OR ventilation) in the databases PubMed, Web of Science and Lilacs. The search included papers published in English, until September 2019, on the effects of positive airway pressure on midfacial growth.

**Results:**

The search strategy identified five studies: two case reports, two cross-sectional studies and one retrospective cohort study. All studies evaluated the long-term effects of a using a nasal mask on the midface in children and adolescents; four showed midface hypoplasia and one no showed difference post- treatment compared to a control.

**Conclusion:**

Most of the studies demonstrated that long-term use of nasal positive airway pressure in childhood/adolescence is associated with midface hypoplasia.

## Resumo

**Introdução:**

O tratamento da apneia obstrutiva do sono com pressão positiva nas vias aéreas em crianças é restrito devido a preocupações de que possa afetar o crescimento da maxila em longo prazo.

**Objetivo:**

Realizar uma revisão sistemática da literatura sobre o impacto em longo prazo do uso de uma máscara de pressão positiva nas vias aéreas na face média em indivíduos em crescimento.

**Método:**

A pesquisa bibliográfica foi realizada em setembro de 2019 utilizando as palavras-chave (*long-term* OR *side effects* OR *longitudinal*) AND (*children* OR *child* OR *preschool* OR *adolescents* OR *adolescent* OR *infant* OR *infants*) AND (*craniofacial* OR *mid*-*face* OR *midface* OR *midfacial* OR *facial* OR *maxillary*) AND (*airway pressure* OR *ventilation*) nas bases de dados PubMed, Web of Science e Lilacs. A pesquisa incluiu artigos publicados em inglês, até setembro de 2019, sobre os efeitos da pressão positiva nas vias aéreas no crescimento médio-facial.

**Resultados:**

A estratégia de busca identificou cinco estudos: dois relatos de casos, dois estudos transversais e um estudo de coorte retrospectivo. Todos os estudos avaliaram os efeitos em longo prazo do uso de máscara nasal na face média em crianças e adolescentes; quatro apresentaram hipoplasia da face média e um paciente não mostrou diferença após o tratamento em comparação com um controle.

**Conclusão:**

A maioria dos estudos demonstrou que o uso prolongado da pressão positiva nas vias aéreas nasal na infância/adolescência está associado à hipoplasia da face média.

## Introduction

Obstructive sleep apnea (OSA) is defined as a respiratory disorder during sleep, characterized by partial or total airway obstruction,^1^ and has a prevalence of 2%–5% in children between 2 and 6 years old.^2^ The main risk factors for OSA in children are hypertrophy of the tonsils, obesity, genetic syndromes and craniofacial alterations.^2^

In children, OSA is a risk factor for behavioral problems, cognitive and learning impairment, and is associated with cardiovascular risk and a poor quality of life.^3^, ^4^, ^5^, ^6^ The treatment of OSA enhances the child's health. The main treatment for OSA in children is adenotonsillectomy surgery,^7^, ^8^, ^9^ which resolves 85%–90% of cases.^10^, ^11^, ^12^ However, some patients still require additional treatment using alternative methods such as corticosteroids,^13^, ^14^ rapid maxillary expansion,^15^ myofunctional therapy,^16^ behavioral measures for weight loss and diet^17^ or positive airway pressure (PAP).^2^

PAP is used as a therapy for children with severe OSA who do not respond to surgery, or have genetic conditions or neurological problems.^18^ The treatment of OSA with PAP in children is restricted due to concerns related to normal facial growth and the risk of the development of maxillary retrognathia over time.^19^ The objective of this study is to undertake a systematic review to investigate the effect of long-term PAP mask on the midface in growing individuals.

## Methods

The systematic review was conducted by two investigators using the databases PubMed, Web of Science and Lilacs, with studies in English published from any date until January 2019. Literature reviews, letters to the editor and studies not directly related to the subject were excluded. Original studies conducted on children and adolescents aiming to evaluate the effects of PAP on the midface were included. The PICO search strategy was used (Table 1).

**Table 1:** PICO strategy.

P: Population or patient	children OR child OR preschool OR adolescents OR adolescent OR infant OR infants
I: Intervention	“airway pressure” OR ventilation
C: Comparison	“long-term” OR “long term” OR “side effects” OR longitudinal
O: Outcome	craniofacial OR “mid-face” OR midface OR midfacial OR facial OR maxillary

The main outcome of interest is clinical, cephalometric and tomographic assessment of the maxilla or midface area after long-term PAP use. The titles and abstracts identified in the search strategy were read by two investigators who selected the papers that met the predetermined eligibility criteria. The main data from each paper were collected in detail and recorded in a standardized table.

## Results

The search strategy retrieved 197 papers from PubMed, 53 from Web of Science and 7 from Lilacs. Among these, only five papers were related to the objective: two case reports, two cross-sectional studies and 1 retrospective cohort study. All studies used a nasal mask. Table 2 presents the selected studies.

**Table 2:** Descriptive studies.

Author	Sample	Study	PAP mask	Follow up	Evaluation	Results
Li et al. (2000)	One case 15 years	Case report	Nasal mask	10 years	Lateral teleradiography (without mensures)	Hypoplasia of midface and skeletal class III;
						No baseline measures were reported.
Villa et al. (2002)	One case 7 years	Case report	Nasal mask	7 years, 10 h/day (mean)	Lateral teleradiography	Maxillary hypoplasia (ANB = −2.7; SNA = 82.4; SNB = 85.1)
						No baseline measures were reported
						After orthopedic treatment (Delaire mask) associated with PAP there was an improvement in cephalometric measurements
Fauroux et al. (2005)	3 groups:	Cross-sectional	Nasal mask	15 months (mean), at least 6 h/day	Clinical evaluation	68% of the sample had global facial flattening
	OSA group, *n* = 16 10 years (mean)					43% had flattening of the forehead; 38% had flattening of the malar area and 28% had flattening in the maxilla
	Neuromuscular disturbance group, *n* = 14 11.8 years (mean)					Concave face was present in 12% of the patients
						Flattening was associated with more than 10 h of use
	Cystic fibrosis group, *n* = 10 15.8 years (mean)					No baseline measures were reported.
Korayem et al. (2013)	2 groups:	Cross-sectional	Nasal mask	6 months (mean), 5.9 h/day (mean)	Lateral teleradiography by CBCT (SN, BaSN, SNA, PP-SN, CoANS, ANS-PNS, U1-PP, Anperp, OLp-A, SNB, ArFome, FoMe, ANB, Wits)	There were no differences between groups in relation to cephalometric measurements
	OSA CPAP group, *n* = 12 9 years (mean)					No baseline measures were reported.
	OSA control group, *n* = 11 10 years (mean)					
Roberts et al. (2016)	2 groups:	Retrospective cohort	Nasal mask	2.6 years (mean), more than 20 h/week	Lateral teleradiography (SNA, SN, ANS-PNS, SN-PP, Ba-S-N, A-SN7, A-SN71, U1-SN, U1-PP)	PAP compliant group showed less positive annual cephalometric changes than the PAP non-compliant group (SNA, ANS-PNS, SN-PP, A-SN7, A-SN71, U1-SN, U1-PP), after adjusting for age, gender, and primary craniofacial diagnosis;
	CPAP compliance, *n* = 50 10 years (mean) CPAP;					
	CPAP noncompliant, *n* = 50 <20 h/week for <6 months 9 years (mean)					The PAP compliant group had more midface retrusion, flaring of the upper incisor and counter-clockwise tipping of the palatal plane than the control.

CBCT, cone beam computer tomography; CPAP, continuous positive airway; OSA, obstructive sleep apnea; PAP, positive airway pressure; ANB (degrees), A-point–nasion–B-point: relative position of mandible to maxilla; A-NPerp (mm), A-point-perpendicular to Frankfort horizontal at N: maxillary projection; ANS-PNS (mm), anterior nasal spine-posterior nasal spine: length of palate; ANS-PNS (mm), length of maxilla; ArGoMe (degrees), articulare–gonion–menton: angle of mandible; A-SN7 (mm), effective maxillary height, vertical distance from anterior maxilla to the SN7 (7° below S-N) line; A-SN7⊥ (mm), effective maxillary length, anteroposterior distance from anterior maxilla to a line perpendicular to the SN7 line; Ba-S-N (degrees), degree of flexure of cranial base; Co-ANS (mm), condylion-ANS: maxillary anteroposterior projection; Go-Me (mm), gonion–menton: length of mandibular body Maxilla-mandible; OLp-A (mm), linear distance between A-point and a line drawn perpendicular to the occlusal plane at sella (OLp); S-N (mm), length of anterior cranial base; SNA (degrees), anteroposterior projection of anterior maxilla relative to anterior cranial base; SNB (degrees), sella–nasion–basion: mandibular anteroposterior projection; SN-PP (degrees), angulation of palatal plane relative to anterior cranial base; U1-SN (degrees), inclination of upper incisor relative to anterior cranial base; U1-PP (degrees), angulation of maxillary incisor to palatal plane; U1-PP (degrees), inclination of upper incisor relative to palatal plane; Wits (mm), distance between perpendiculars to occlusal plane at Points A and B.

### Case reports

Li et al. (2000),^20^ reported the case of a 15 year-old boy with OSA and obesity, treated since five years of age with nasal CPAP. Due to the long-term utilization of a nasal mask, the child showed midface hypoplasia with a concave face, but no previous cephalometric exam was presented. They concluded that the chronic use of a facial mask for home ventilation in children should be associated with regular evaluation of maxillomandibular growth.

Villa et al. (2002),^19^ reported a clinical report of a child using BIPAP from a newborn to the age of seven-years old, and reported that the child showed midface hypoplasia. No cephalometric exam prior to the BIPAP was reported, and orthopedic treatment with a Delaire mask (maxillary protraction device) combined with a nasal ventilation mask seemed to improve the midface retrognathia.

### Cross-sectional studies

Fauroux et al. (2005),^21^ conducted a cross-sectional study to quantify the side effects of using a nasal mask for PAP in children. The sample was comprised of patients with OSA (*n* = 16), neuromuscular disorders (*n* = 14) and cystic fibrosis (*n* = 10), with 15 months of PAP use. The interventions included clinical evaluations and reported global facial flattening in 68% of patients. Maxillary flattening was present in 28% of patients and 12% had a concave face. No baseline measures were presented in the study. Flattening was associated with more than 10 h/night of PAP use, but no correlation was observed with age, daily or cumulative use. They concluded that the prevalence of facial side effects is clinically relevant in children using PAP.

Korayem et al. (2013),^22^ conducted a cross-sectional study using a control group without CPAP. They did not identify differences in cephalometric parameters between groups in 6 months of follow up. They did not observe an association between anteroposterior maxillary positioning and the duration of therapy or hours of CPAP use compared to a control group. They did not analyze cephalometric parameters before treatment.

### Retrospective cohort

Roberts et al. (2016),^23^ conducted a retrospective cohort study, with a large sample size (*n* = 100) and with a mean followup of 2.57 years, and compared groups of compliant and non-compliant CPAP users. They observed that individuals who used CPAP demonstrated worse annual cephalometric changes compared to individuals in the non-compliant group, after adjusting for age, gender, and primary craniofacial diagnosis. CPAP compliant individuals had more midface retrusion, flaring of the upper incisor and counter-clockwise tipping of the palatal plane than the control. It is the only study that used parameters from before treatment.

## Discussion

All the studies observed midface hypoplasia in children and adolescents who used nasal PAP mask in the long term, except for the study by Korayem et al. (2013).^22^ Only one study used measurements from before the PAP treatment associated a control group.^23^ The other studies reported the frequency of alterations after PAP, including syndromic patients,^21^ which is a risk factor for the facial growth, or analyzed characteristics after PAP compared to a control group, without considering any baseline characteristics.^22^ In some cases there were syndromic patients in control groups, that are other risk factors for alterations on middle-face growth. The two case reports showed alterations after treatment, but did not describe baseline characteristics.^19^, ^20^ All studies involved PAP use of more than 1 year, and most showed good PAP compliance.

The only study disagreeing with the others had limitations and the results should be carefully interpreted. This was a cross-sectional study, using only values post-treatment, not considering baseline characteristics, with a relatively small sample size. Most studies about the effect of PAP on the midface have low methodological quality and more studies are required to reach any valid conclusion.

PAP treats OSA by the application of positive pressure using an external mask around the nasal interface, applying substantial pressure on the tissue and adjacent bones in the opposite direction to sutural growth.^20^ It thus possibly restricts growth and leads to midface hypoplasia, a concave profile and Class III malocclusion.^19^ The orthopedic changes in facial bones by application of forces for a long period has been the basis of dentofacial orthodontic and orthopedic treatment.^24^ In Orthodontics, effects of 500 g forces on the nasomaxillary complex using a headgear, for a minimum period of 10 h, restricts the anterior downward maxillary displacement.^25^

In the concept of Orthodontics, bone movement occurs by the interaction of three factors: time of force application, frequency of force application, and intensity of force application.^26^ PAP is a treatment that requires good compliance, ideally with utilization throughout the sleep period (ideally 10 h), with a daily frequency and the mask firmly connected to avoid air escape. Within this context, there is an interaction between these factors that interfere with the direction of bone growth. Thus, it is important to evaluate these three orthodontic parameters during the treatment to assess any changes in bone structure. Faroux et al.^21^ observed that changes in facial flattening were associated with the use of PAP for more than 10 h/night.

Li et al.^20^ suggested that extended force application on the developing facial skeleton may cause harmful effects on growth or worsen existing problems. Li et al.,^20^ Villa et al.^19^ and Roberts et al.^23^ suggest that individuals should receive regular maxillomandibular evaluations (at least yearly), and databanks should be obtained from such evaluations.^20^ It should also be noted that midface retrusion may potentiate the OSA, often requiring higher PAP pressures and surgical bone correction.^23^

It is vital that there is monitoring by an orthodontist to evaluate, prevent and correct any complications related to facial growth arising from the use of PAP. If any problems are identified there are a number of possible options such as: reducing the force of the mask pressure on the bones; checking the mask fit; or modifying the type of mask, perhaps using an intranasal mask to try to reduce the impact on bone structure (studies are still required to evaluate this option). The 2002 study by Villa^19^ treated the facial deformity using a facial orthopedic appliance (Delaire mask) concomitantly with the use of PAP and observed an improved facial profile. This facial mask applies a force opposite to the PAP, redirecting the forward maxillary growth.

Unfortunately, there are few studies about the effect of PAP mask pressure on midface growth, there is a lack of randomized prospective studies, in most of studies no baseline characteristics of sample are evaluated, and in some case syndromic patients were included, which may impact on growing of midface. More studies evaluating the possible side effects of long-term PAP use and of ways to prevent them are clearly needed.

## Conclusion

Most studies indicated that the long-term use of PAP impacts midface growth in children with OSA, resulting in hypoplasia. There is a lack of studies using a strong methodology to evaluate facial growth in children using PAP.

## Funding

Associação de Incentivo à Pesquisa (10.13039/501100006303AFIP), Coordenação de Aperfeiçoamento de Pessoal de Nível Superior (10.13039/501100002322CAPES) – provided material and financial support. Number: 88882.430440/2019-01.

## Conflicts of interest

The authors declare no conflicts of interest.
